# Are viral-infections associated with Ménière’s Disease? A systematic review and meta-analysis of molecular-markers of viral-infection in case-controlled observational studies of MD

**DOI:** 10.1371/journal.pone.0225650

**Published:** 2019-11-22

**Authors:** Nicholas John Dean, Christopher Pastras, Daniel Brown, Aaron Camp

**Affiliations:** 1 The University of Sydney, Sydney, NSW, Australia; 2 School of Medical Sciences, The University of Sydney, Sydney, NSW, Australia; 3 School of Pharmacy and Biomedical Sciences, Curtin University, Bentley, WA, Australia; Clinica Universidad de Navarra, SPAIN

## Abstract

Despite considerable research, it remains controversial as to whether viral-infections are associated with Meniere’s Disease (MD), a clinically heterogeneous set of chronic inner-ear disorders strongly associated with endolymphatic hydrops. Here, we investigated whether viral-infections are associated with MD through a systematic review and meta-analysis of observational clinical studies using molecular-diagnostics. Eligible for inclusion were case-controlled studies which ascertained molecular-determinants of past or present viral-infection through either viral nucleic acids or host serological marker in MD cases and non-MD controls. Across online databases and grey literature, we identified 210 potentially relevant articles in the English language, from which a total of 14 articles fully satisfied our eligibility criteria such that meta-groups of 611 MD-cases and 373 controls resulted. The aggregate quality of the modest-sized (14 studies) body of evidence was limited and varied considerably with regards to participant selection, matching, and ascertainment(s) and determinant(s) of viral-infection. Most data identified concerned the human cytomegalovirus (CMV), and meta-analysis of eligible studies revealed that evidence of CMV-infection was associated approximately three-fold with MD compared to controls, however the timing of the infections was indeterminate as the pooled analyses combined antiviral serological markers with viral nucleic acid markers. No association was found for any of HSV-1, -2, VZV, or EBV. Associative analyses of any viral species not aforementioned were precluded by limited data, and thus potential associations between other viral species and MD, especially other than *Herpesviridae*, are yet to be characterised. Overall, we have found a small association between CMV-infection and MD, however it is to be determined for what sub-groups of MD this finding may be relevant, and ideally the reported association remains would be reproduced by a greater volume of higher quality evidence.

## Introduction

Meniere’s Disease (MD) is a relatively rare, considerably heterogeneous collection of chronic inner-ear disorders involving a characteristic triad of symptoms: recurrent sporadic vertigo, tinnitus, and fluctuating sensorineuronal hearing loss (SNHL) of low-medium frequencies [1–8]. MD presents with an inner-ear histopathology termed endolymphatic hydrops (EH), itself a distension of the fluid-filled membranous component of the labyrinth, however EH alone does not necessarily entail a clinical diagnosis of MD [9]. Because EH is present in conditions other than MD, MD is itself a particular variety of a more general phenomenon of hydropic ear disease [10, 11]. Emerging etiological accounts of MD are multifactorial, with a range of clinical subgroups now described [2–8]. While most of the incidence of MD is considered sporadic, about a third of those with MD exhibit evidence of pro-inflammatory cytokine mediated immune dysfunction [3, 5, 12]. About 5% of cases of MD show evidence of heritability, and in such cases NF-κB mediated inflammation appears influential [13]. Overall, several lines of epidemiological evidence suggest that between various MD clinical sub-groups, inflammatory signatures and immunotype changes in the inner-ear are common [12, 13].

Given that immunopathology of the inner-ear is of a considerable prevalence in MD, it is important to determine relevant etiological factors. As early as the 1960s, it was hypothesized that infectious agents such as viruses might contribute to the development and/or severity of various pathophysiological processes in MD [14–17]. However, whether viral-infections are in fact relevant to MD is controversial. In a 2009 review of non-randomized quantitative descriptive studies, Gacek concluded that MD probably simply *is* the manifestation of virally induced vestibular neuropathy [18]. And as of 2012, Greco and colleagues stated that antiviral pharmacotherapy had “virtually eliminated” the use of surgical management for MD [12]. From that perspective, it appears that viral-infections are undoubtedly relevant to MD. By contrast, as of 2008, drawing from an evidence base virtually the same as above, Oliviera and colleagues disputed there being any reliable evidence of a viral etiopathology in MD [19]. And more recently, Mirza and Gokhale judged that antiviral pharmacotherapy has had “no role in the treatment of MD” [20]. So, on the other hand it appears that there is a lack of evidence for any association between viral-infection and MD. Based on such discrepancies, there appears to be a lack of a clear relationship between viral-infection and clinically diagnosed cases of MD. Further, despite this lack of consensus, to the best of our knowledge, whether viral-infections are associated with MD has not been explored in a published systematic review and meta-analysis.

Exploring whether viral-infections are associated with MD involves, at the single study level, that at minimum it be shown that viral-infections are of significant prevalence in those with MD. Of course, investigations of associations between infectious pathogens and diseases in observational clinical studies must take into account that certain species of virus familiar to neurotology such as the *Herpesviridae* are particularly seroprevalent in humans, and thus it is preferable that case-controlled studies are used for such etiological investigations [21]. A prevalence of viral-infection can be ascertained through various methods, including classical virologic techniques such as transmission-electron microscopy (TEM) and viral cultures, or to make a somewhat arbitrary distinction, a range of methods that afford higher sensitivity, specificity and throughput, broadly referred to as ‘molecular-diagnostics’ [22, 23]. Here, we took molecular-markers as the ‘gold-standard’ not only for technical reasons, but also due to the following methodological considerations. A potential relationship between viral-infection and MD has in the past been investigated in clinical settings using methods neither specific nor especially sensitive, which may in part underlie inconsistent reports as to whether or not virions are more often isolatable from those with MD than controls. In particular, analyses of the presence of virions in the MD inner-ear have largely relied on TEM. Of those, in [24–26], Gacek describes virions and associated viral reproductive machinery in post-mortem inner-ear sections of MD vestibular ganglia. By comparison, Palva and colleagues’ discrepant observations when using effectively the same TEM methodology augmented by viral culture [27]. Further, and perhaps most importantly, Wackym and colleagues’ also found a lack of virion involvement in MD using TEM, while also noting the presence of particles that “morphologically mimic[ked] viruses” but were much more likely to be non-pathological, regularly occurring cellular components [28]. Clearly, a systematic synthesis would be necessary to resolve such discrepancies, but the nature of the aforementioned TEM data does not provide much in the way of intercomparable quantifications. Overall, while TEM virologic studies of MD have been useful in defining exploratory hypotheses, the nature of such studies is sub-optimal for quantitative epidemiological syntheses, and with regards to the actual body of evidence consisting in those studies, there are several concerns as to both between-study qualitative differences and the classical virologic methods themselves. Overall, case-controlled clinical observational studies employing molecular diagnostics via either serological or nucleic acid biomarkers are probably the best available source for investigating whether or not particular viruses, or indeed other infectious agents, are associated with MD.

Exploring whether viral-infection is associated with at least some cases of MD is important not only in further understanding the etiology of MD, but it may also provide comment on the use of antiviral pharmacotherapeutics in MD, which thus far have seen little success in clinical trials [29]. Indeed, viral-hypotheses of MD have been implicitly used in studies of anti-viral pharmacotherapy. While a number of studies have investigated antiviral pharmacotherapy for MD, only two have been identified as randomized-controlled trials, and in those, sub-optimal reporting has precluded a meta-analytic estimation of overall effects, though neither individual study identified any substantial therapeutic efficacy [29]. With regards to the respective results, Guyot and colleagues found that intratympanic ganciclovir afforded no improvement over an intratympanic delivery of sodium-chloride solution with regards to delaying surgical intervention, while Derebery and colleagues found that oral famciclovir may have reduced hearing fluctuation, but not vertigo or dizziness [30, 31]. More recently, Gacek reported hearing improvements in 38% of 31-patient MD cohort, with a further reported complete control of vertigo in those 12 of the patients with improved hearing, however this work is limited by a lack of placebo, randomization or other control measure [18]. Overall, few published studies have examined the efficacy of antiviral pharmacotherapeutics for MD, and no strong indication for their use has been identified. Despite unpromising results, it should be recognised that a lack of evidence for pharmacotherapeutic efficacy does not constitute suitable grounds by which to dismiss narrower, well-targeted viral-hypotheses of MD and therefore any future studies of antiviral pharmacotherapy in MD would probably be well-informed by a systematic review of viral-infection in MD.

While viral-infections could be relevant to some cases of MD, findings between studies are discrepant and thus far that hypothesis remains controversial. Thus, we asked whether MD is associated with evidence of past or current viral-infection. We answered this through a systematic review and meta-analysis of clinical observational studies which had employed molecular ascertainments of viral-infection on a case-controlled basis.

## Methods

Two reviewers (ND and AC) independently undertook (i) a transparent study inclusion/exclusion procedure (search, selection, and data extraction), and (ii) a structured appraisal of study quality involving an identification of study design with a subsequent appraisal of methodology. All reviewers contributed to the study design and other components together. The review protocol was not pre-registered with PROSPERO. In accord with our aims to perform a systematic review, we followed PRISMA-P guidelines (see S1 for a completed PRISMA checklist (2009 version)) [32]. In accord with our aims to perform a meta-analysis of observational epidemiological data, also followed MOOSE guidelines, and these guidelines were our transparent reporting items (see S2 for the completed checklist) [33].

### Search strategy

We conducted primary and secondary searches to identify any potentially relevant peer-reviewed published evidence. We did not consider evidence that had not been published, nor abstracts without a full-text. The primary (systematic) search was of the following online databases: MEDLINE (OvidSP; 1946–2019), EMBASE (OvidSP; 1947–2019), Web of Science (Clarivate Analytics; 1900–2019), and SCOPUS (Elsevier; 1788–2019). Our search terms were applied to subject headings (MeSH), keywords, titles, abstracts, and topics and were as follows: [(meniere* OR endolymphatic hydrops) AND (*virus* OR *viral* OR *virol*). The (*)s refer to ‘unlimited wild-card modifiers’ utilized to account for between text terminological variations including the use of plurals, hyphenated terms, terms in different arrangements and spelling variations. We did not apply date limits to our searches. At the level of the respective databases, we filtered for articles written in the English language, and filtered out any and all reviews, case-reports (without control groups), case-series (without control groups). The filtered results were exported from each database and collated in reference management software where any duplicates were deleted. We enacted forward tracking (auto-updates) of the online database searches for the entirety of the time in which the manuscript was being written. The secondary search consisted in hand searches of reference lists of studies obtained from the primary search and Google Scholar search conducted in August 2019. Within Google Scholar, the default search settings were used and the search terms were: ((viral OR virus) AND (meniere’s disease OR endolymphatic hydrops).

### Definitions

Diagnostic criteria for MD have undergone several revisions throughout the time period from which included studies were selected from [34–36]. To minimize bias based upon a preference for any particular diagnostic criteria, we simply required that potentially eligible studies cited a recognized diagnostic criteria (and was not seen to be then clearly contradicted in the study), or else it was explicitly stated how the diagnosis/classification of MD was ascertained and this was deemed by all four authors of this paper to be in sufficient overlap with criteria from [34–36]. Our use of the Newcastle-Ottawa-Scale (NOS) (described in the section ‘Systematic rating of studies’) for critical appraisal of study methodologies included an appraisal of a given studies’ disease definition.

With regards to the verification of evidence of viral-infection in MD and control subjects, molecular ascertainments were distinguished a priori between those that aimed to (i) detect the presence of specific viral species via their nucleic acids (during either active or latent infection) as opposed to (ii) detect evidence of infection at an indeterminate time through immunoglobulin antiviral antibodies. The former measurements are herein referred to as ‘direct’ and the latter as ‘indirect’. This arbitrary distinction between ‘direct’ (nucleic acid determinant) and ‘indirect’ (serological determinant) ascertainment was important as we hypothesized that this factor may introduce a significant degree of heterogeneity between the included studies, and any such associations would not be valid.

### Study eligibility requirements

We initially screened articles for inclusion or exclusion at the level of their title and abstract. If it was unclear whether an article should be included based on its title and/or abstract, the article was kept for full-text review. ND and AC aimed to include or exclude studies based only on the criteria detailed in Table 1. There were four criteria, and these were loosely based on a ‘PICO’ inclusion/exclusion template.

**Table 1:** Eligibility criteria.

Factor	Requirements
Study-design	Case-controlled (corresponding to at least NHMRC level III-3) observational studies that ascertained a history of viral-infection (indeterminate time-scale) with molecular verifications (refer to the ‘critical appraisal of included studies’ section).
Participants	A pre-established (clinical) diagnosis of MD or else indication of a systematic, consistent inclusion/exclusion process MD group participants (see ‘definitions’ above).
Ascertainment(s) of infection	Direct ascertainments: nucleic acid amplification (PCR and optimization variants) or in-situ hybridization. Indirect ascertainments: immunoassays. For up-to-date reviews of specific molecular methods for diagnostic virology, see [22, 23].
Determinant(s) of infection	Direct determinants: viral nucleic acid(s) (DNA or RNA). Indirect determinants: immunoglobulin antiviral antibodies. Articles assessing infection by any viral pathogen known to the authors were eligible for inclusion, and otherwise The National Centre for Bioinformatic Information (NCBI) viral genome database would be checked in the case of any very uncommon species [37].

### Data extraction

For all eligible full-text studies, data was extracted into (duplicate) semi-structured spreadsheets by two independent reviewers (ND and AC). We did not attempt to contact respective authors if data was not fully reported in published articles. The data was coded as follows: author(s), year of publication, country of study, number of participants in each group and in total, MD group definition, control group definition, power-analyses, method of ascertainment of viral-infection and the determinant used, viral species investigated, average age of MD participants. The separate data-extractions were merged into a single spreadsheet. For each study, we developed 2-by-2 case-control contingency tables indicating proportion in each respective group who were positive and negative for a given determinant.

### Critical appraisal of included studies

We systematically performed critical appraisals of the included studies in order to assess the risk-of-bias of included studies alongside the ‘quality’ of a given studies’ methodological design. Many tools are available for critically appraising study methodologies and assessing the risk of bias of studies included in systematic reviews. With specific regards to systematic reviews of observational studies of etiology, as of yet there are no tools recognized as obviously preferential (for reviews on this matter, see [38, 39]). Thus, we used the Newcastle-Ottawa Scale (NOS) for a systematic appraisal of the ‘quality’ of each included studies’ methodology, a tool which is simple to both apply and interpret [40]. We defined high-quality evidence as that which corresponded to a low ‘risk of bias’, and vice versa. Each studies’ classification in NHMRC evidence hierarchy (inherent to the study design) was also indicated [41]. With regards to the adaptation of the NOS for the present work, the ‘selection’, ‘control’, and ‘exposure’ assessment structure of NOS was not altered and so studies would score up to four, two, and three stars in each domain of NOS. The content of the exposure assessment was adapted to the context of the research question that exposure was defined as “molecular evidence of past and/or present (and either active or latent) viral-infection”. Specifically, for item E1 concerning the “ascertainment of exposure”, the use of a “structured interview where blinded to case/control status” was exchanged for the use of a “methodologically sound, recognized molecular diagnostic approach”, and the ascertainment of exposure via a “secure record (e.g. surgical records)” was omitted from the scoring. Finally, we noted (i) whether studies indicated a priori power analyses, and (ii) whether studies with <20 participants in any group used equal sample sizes for each group or else provided a justification for group sizing.

### Data synthesis and meta-analysis

Data and meta-analysis were conducted using R (v3.6.0). For the purposes of meta-analysis, we also used the “metafor” and “metaviz” extensions for R (see [42] for “metafor”; see [43] for “metaviz”). It was determined a priori that all pooled analyses would be segregated at the level of viral species, but not particular strains thereof (see [37] for details on this taxonomy).

For a particular viral species, where infection of the host was assessed in more than one study, we attempted to extract contingency tables. Where contingency tables were extractable in full, we calculated unadjusted odds ratios (ORs) for MD given evidence of viral-infection at the 95% confidence interval (C.I.) level. The unadjusted ORs underwent logarithmic transformation (‘logOR’) for displaying the pooled and summary effects. Where contingency tables involved null-data, we applied the Anscombe-Haldane continuity correction (linear transformation of +0.5) as per the Cochrane handbook [44, 45]. Where a study assessed the evidence for viral-infection through more than one ascertainment/determinant pairing such that there were multiple contingency tables from the same study for the same viral species, ORs were calculated separately for each ascertainment/determinant pairing so as to ensure that any subsequent pooled analyses would not count any participants more than once. Thus, for a study with multiple measures of viral-infection, each ascertainment-determinant pairing was reported separately and no pairing was used in combination for pooled effect size calculations. Due to the variability of study contexts, pooled effect sizes (pooled ORs) were determined under random-effects (RE) assumptions, i.e. using inverse-variance (*τ*^2^) estimators, which themselves minimize the variance of the weighted average [42]. For each viral species, overall effects were first determined using the Der-Simonian Laird (DL) *τ*^2^ estimator, a model of RE which is usually less sensitive to variance [42]. If the 95% C.I.s calculated under the DL model did not overlap with chance, overall effects would then be recalculated under the usually more conservative restricted maximum likelihood *τ*^2^ estimator (REML) [42].

Heterogeneity for the overall effects was determined through the chi-squared statistic (Cochran’s *Q*; 95% probability cut-off), which determines a statistical significance of between-study differences. This was used in conjunction with Higgin’s *I*^2^, which determines the percentage of the variance of the pooled effect that can be attributed to heterogeneity. We defined ‘significant’ heterogeneity as (p-value) as p<0.05 (Cochran’s *Q*) and ‘substantial’ heterogeneity as *I*^2^>25% (Higgin’s *I*^2^), and either/or would be investigated. For those overall effects where significant heterogeneity was present, the mode of ascertainment (direct or indirect) was then used as a moderator variable in a mixed effects sub-group analysis. The result of such an analysis provides an indication of how much of the variance between studies can be attributed to differences in measuring signatures of viruses themselves at the time of study as opposed to measuring host antiviral antibodies specific for viruses which may no longer inhabit the host.

Where overall effects did not overlap with chance under *both* DL and REML estimators, we conducted leave-one-out sensitivity analyses to try to identify low-quality (NOS) studies or else outliers which might have biased the overall effect. Further for those overall effects significant under both DL and REML estimators, we gauged potential power/sample size effects (small-study publication biases) through funnel plots, as plots of variability against effect size are usually skewed or else asymmetrical in the presence of publication and related biases, and such biases are more likely in smaller studies [46]. Our funnel plot analyses involved four contour-enhanced funnel plots of both standard error and sampling variance metrics, along with their inverses, in order to potentially gauge any clustering of lower and/or higher-powered studies [46]. The data for the funnel plots underwent Duval and Tweedie’s trim-and-fill adjustment [47]. Next, Egger’s meta-regression test for funnel plot asymmetry was computed for both DL and REML models, whereby standard error is used as a predictor for detecting funnel-lot asymmetry. Finally, the authors observed the funnel plots in order to note any biases that may have gone undetected by statistical testing.

## Results

### Study selection

**Fig 1:**
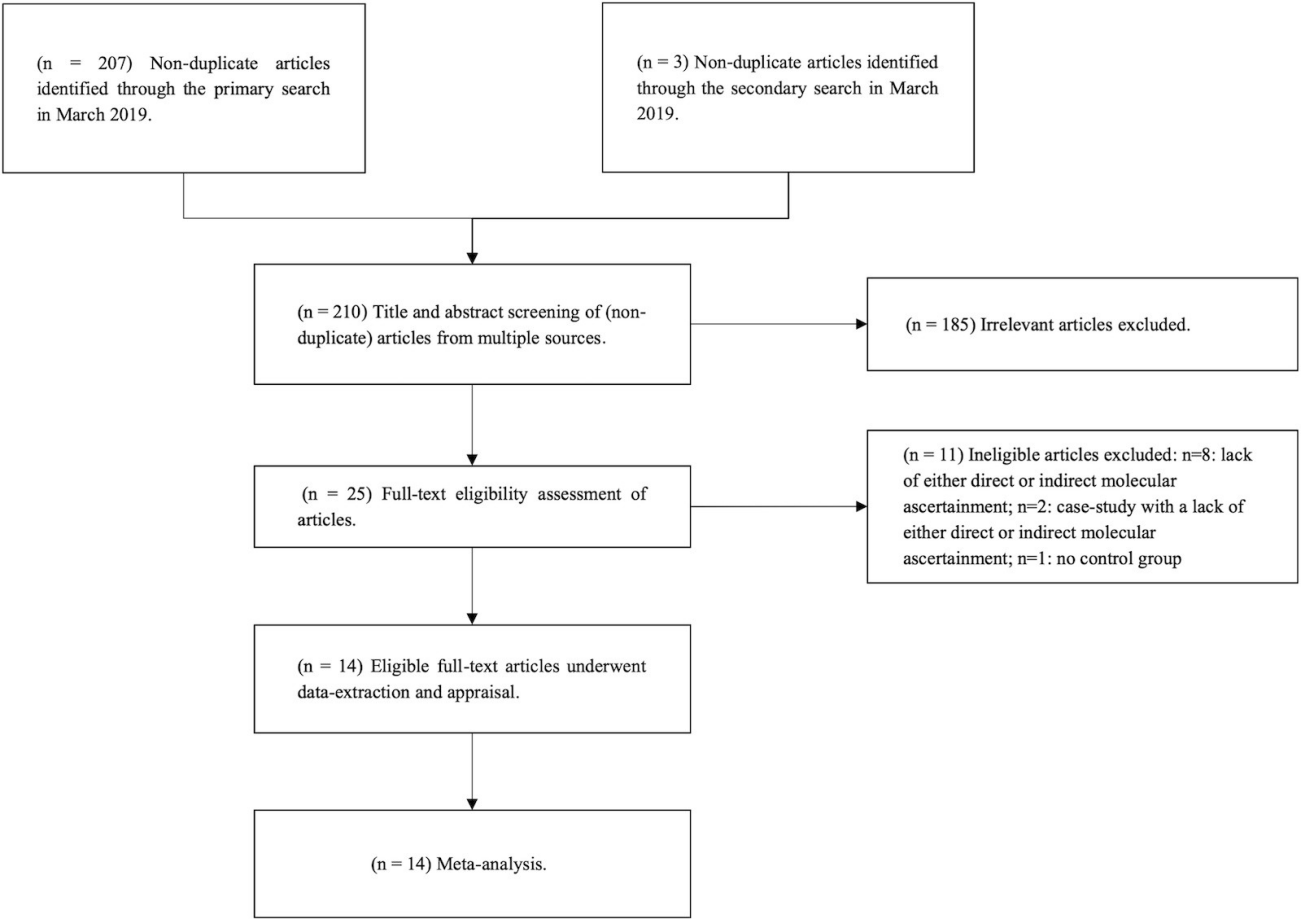
PRISMA flow-diagram of the search strategy and study selection process.

A total of 14 publications met the criteria for our review [48–61]. Our searches of MEDLINE, EMBASE, Web of Science, and SCOPUS yielded 207 non-duplicate articles, and our supplementary search yielded an additional 3 non-duplicate articles, with the searches together constituting a total of 210 unique articles. After the screening of those 210 articles at the level of title and abstract, 183 records were excluded for irrelevance. After the full-text screening of 27 remaining articles, a further 13 were excluded for reasons detailed in Fig 1. Thus, after screening, 14 full-text studies satisfied our eligibility requirements and were included for data extraction, critical appraisal and data synthesis.

### Characteristics of studies

The characteristics of the included studies are summarised below in Table 2. Across all 14 studies, a total of 611 cases of MD ($\bar{x}=44$) were matched to 373 controls ($\bar{x}=27$), corresponding to an average of eight cases of MD for every five controls. Due to reasons including methodological variance, not all studies reported the average age of their participants. The average age of MD participants from studies who did report such data was 52.6 years, however it should be noted that not all of the included studies were age-matched (see Table 3.) [49, 50, 54, 55, 56, 60, 61]. A range of methodological characteristics were identified in the studies. Seven studies directly ascertained viral-infection through nucleic acids: six of those through PCR-variants and one utilized in-situ hybridization. Eight studies indirectly ascertained viral-infection through host antiviral immunoglobulin antibodies: six of those studies measured these antibodies through ELISA, one through RAST, and one through IFA. Only Takahashi and colleagues utilized both direct and indirect ascertainments of viral-infection, although this was only the case for HSV-1 and VZV and not HSV-2 or HCMV [56]. The included studies were published over a period of 21 years between 1987 and 2008.

**Table 2:** Summary characteristics of the included studies.

Study	Ascertainment	Determinant	Viral Species									
			1	2	3	4	5	6	7	8	9	Other(s)
Arnold and Niedermeyer, 1997 [49]	ELISA	antiviral IgG (serum, perilymph)	HSV-1	CMV			EBV		MV			-
Bergstrom et al., 1992 [50]	ELISA	antiviral IgG (serum)	HSV-1	CMV	HSV-2	VZV			MV			-
Calenoff et al., 1995 [51]	RAST	antiviral IgE (serum)	HSV-1	CMV	HSV-2		EBV					-
Pyykko et al., 2008 [53]	ELISA	antiviral IgG (serum)	HSV-1	CMV	HSV-2	VZV	EBV	AV		I-A	I-B	Yes
Selmani et al., 2004 [54]	ELISA	antiviral IgG (serum)	HSV-1	CMV	HSV-2	VZV	EBV	AV		I-A	I-B	-
Selmani et al., 2005 [55]	ELISA	antiviral IgG (serum)	HSV-1	CMV	HSV-2	VZV		AV				-
Williams et al., 1987 [60]	ELISA	antiviral IgG (serum)	HSV-1	CMV		VZV						Yes
Takahashi et al., 2001 [56]	IFA	antiviral IgG (serum)	HSV-1	CMV	HSV-2	VZV						-
	PCR	viral DNA in peripheral blood mononuclear cells (in-vivo)										
Arenberg et al., 1997 [48]	N-PCR	viral DNA in ES (biopsy)		CMV								-
Gartner et al., 2008 [52]	N-PCR	viral DNA in SG (biopsy)	HSV-1		HSV-2	VZV						-
Vrabec, 2003 [57]	RT-PCR	viral DNA in SG (archives and biopsy)	HSV-1		HSV-2							-
Welling et al., 1994 [58]	PCR	viral DNA in ES (biopsy)	HSV-1	CMV	HSV-2	VZV						-
Welling et al., 1997 [59]	PCR	viral DNA in ES (biopsy)	HSV-1	CMV	HSV-2	VZV						-
Yazawa et al., 2003 [61]	ISH	viral DNA in ES (biopsy)	HSV-1	CMV	HSV-2	VZV	EBV					-
**Relative Frequency of Ascertainment Across All Studies (%)**			93%	86%	79%	71%	36%	21%	14%	14%	14%	14%

AV, adenovirus; CMV, human cytomegalovirus; ELISA, enzyme-linked immunosorbent assay; ES, endolymphatic sac; EBV, Epstein-Barr virus; HSV-1/2, herpes simplex virus 1/2; I-A/B, influenza A/B; IFA, indirect fluorescent antibody test; ISH, *in-situ* hybridisation; MV, Measles virus; N-PCR, nested polymerase chain reaction; PCR, polymerase chain reaction; RAST, radioallergosorbent test; RT-PCR, real time fluorescence polymerase chain reaction; SG, Scarpa’s Ganglion.

**Table 3:** Summary appraisal of the included full-text studies.

Study	Country	Design	Sample Sizes (MD, C)	MD Group Definition	Control Group Definition	Newcastle-Ottawa Scale (S/C/O)
Arenberg et al., 1997 [48]	USA	CC	9, 9	AAO-HNS (1985).	Vestibular schwannoma patients.	★★☆☆/☆☆/★★★
Arnold and Niedermeyer, 1997 [49]	Germany	CC	7, 9*	No serviceable hearing, disabling vertigo, tinnitus.	Otosclerosis (n = 7) and pediatric cochlear implant (n = 2) patients.	★★☆☆/☆☆/★★★
Bergstrom et al., 1992 [50]	Sweden	CC	21, 21	Fluctuating hearing loss, vertigo, tinnitus, aural fullness.	Healthy individuals with no history of ear disease, vertigo, or tinnitus.	★★★☆/★★/★★★
Calenoff et al., 1995 [51]	USA	CC	10, 10	Unilateral fluctuating SNHL, vertigo, tinnitus, coincident aural fullness of the affected ear; symptoms for at least 3 years.	Individuals with significant allergic rhinitis with RAST-verified sensitivities to common allergens.	★★☆★/★★/★★★
Gartner et al, 2008 [52]	Switzerland	CC	7, 56**	AAO-HNS (1995).	Individuals with no history of facial weakness or MD.	★★☆★/☆☆/★★☆
Pyykko et al., 2008 [53]	Finland	CC	158, 43***	NS.	Vestibular schwannoma (n = 22), tinnitus (n = 10) and otosclerosis (n = 11).	★☆☆☆/☆☆/★★☆
Selmani et al., 2004 [54]	Finland	CC	159, 26	AAO-HNS (1995).	Vestibular schwannoma patients.	★★☆☆/☆☆/★★☆
Selmani et al., 2005 [55]	Finland	CC	109, 26	AAO-HNS (1995).	Vestibular schwannoma patients.	★★☆☆/☆☆/★★☆
Takahashi et al., 2001 [56]	Japan	CC	28, 100****	Audiometrically documented fluctuating SNHL, intermittent "whirling" vertigo, tinnitus.	Healthy individuals (n = 50) and pregnant women (n = 50).	★★★☆/★☆/★★☆
Vrabec, 2003 [57]	USA	CC	35, 19	AAO-HNS (1995).	Body donors with ‘unknown’ otological history.	★★☆☆/☆☆/★★★
Welling et al., 1994 [58]	USA	CC	22, 11	Audiometrically documented fluctuating SNHL, tinnitus, intermittent "true" vertigo.	Vestibular schwannoma patients.	★★☆☆/☆☆/★★☆
Welling et al., 1997 [59]	USA	CC	11, 11	Fluctuating SNHL, intermittent vertigo lasting 20minutes-24hours, tinnitus, unilateral coincident aural fullness.	Vestibular schwannoma patients.	★★☆☆/☆☆/★★☆
Williams et al., 1987 [60]	USA	CC	25, 25	Audiometrically documented fluctuating SNHL, intermittent vertigo, tinnitus for at least four years.	Individuals who were not acutely ill nor experiencing any symptoms of inner-ear disease.	★☆☆★/★☆/★☆★
Yazawa et al., 2003 [61]	Japan	CC	10, 7	SNHL, intermittent vertigo, tinnitus and "positive glycerol test and/or dominant negative summating potential upon the electrocochleography".	Autopsy specimens without a history of premortem ear disease (n = 6) and vestibular schwannoma (n = 1).	★★☆★/☆☆/★☆★

AAO-HNS, American Academy of Otolaryngology-Head and Neck Surgery; C, control; CC, case-controlled; DL, Der-Simonian Laird MD, Meniere’s Disease; NHMRC, National Health and Medical Research Council (Australia); S/C/O, selection/comparability/outcome assessment; SNHL, sensori-neuronal hearing loss.

*Composition = 7+2.

**composition = 22 (Scarpa’s Ganglion) + 34 (geniculate ganglion)

*** composition = 22 (vestibular schwannoma) + 11 (otosclerosis) + 10 (tinnitus)

**** composition = 50 (healthy) + 50 (pregnant women).

The included studies investigated infection by a range of viral species. At least one member of the *Herpesviridae* genus was assessed in each study, and in particular, the species HSV-1, CMV, HSV-2, and VZV were assessed in the majority of included studies (93%, 86%, 79%, 71%), while EBV, AV, MV, I-A, I-B, and other mostly non-*Herpesviridae* species were each assessed in less than 50% of the included studies (36%, 21%, 14%, 14%, 14%, and 14% respectively). The nature of the determinants of viral-infection varied between the included studies. Of the seven studies that ascertained infection through viral nucleic acids, four targeted the endolymphatic sac (ES) [48, 58–61], two targeted Scarpa’s Ganglion (SG) [52, 57], and one targeted the periphery (serum leukocytes) [56]. For studies that made indirect ascertainments of viral infection, six of eight studies measured serum IgG only [47, 50–53], one measured both serum and perilymphatic IgG [49], and a single study measured serum IgE [51].

### Critical appraisals of included studies

The results of our systematic appraisal of studies using the NOS are presented below in Table 3. All studies attained at least two out of three stars with in rating their assessment of exposure, while almost all studies performed poorer with regards to both participant selection and control matching. The mean attainments for each respective selection/control/outcome component of the NOS were as follows: 2/4, 1/2, and 2/3. The mean overall ascertainment was 5/9. Reported MD selection criteria showed qualitative variation across studies, with 50% of studies stating adherence to a particular recognized diagnostic guideline. While all studies reported that MD was excluded in the control groups, only four studies either used near enough or obviously healthy participants. Two of these studies stated that their participants were both healthy beyond merely being free of a history of inner-ear disease [50, 56]. In one study, it was stated that the control group was not ‘acutely’ ill nor experiencing symptoms of inner-ear disease, but it was not made explicit whether controls were known to have any chronic diseases [60]. Another study stated that there was no history of MD or facial weakness in its control group but did not mention whether participants were known to have any other conditions whether acute or chronic [52]. The most commonly reported health issue in the control groups was vestibular schwannoma (50% of all control groups). Other conditions identified in the control groups were otosclerosis (2/14), allergic rhinitis (1/14), tinnitus (1/14) [. One study reported that its control group had an unknown otological history [57].

With regards to the a priori statistical design of the studies, six studies used at least 1:1 matching, while in the remaining eight studies it was either not made explicit or else still unclear as to the justification for the number of participants and controls respectively. None of the included studies reported a priori power analyses. Finally, in eight of the fourteen studies, at least the case or control group had <20 participants, and in six of those eight studies, the control groups were not of equal size. All study designs were level III-3 on the NHMRC level of evidence scale, with an absence of nested case-controlled studies (level II). On the basis of study design alone, the body of evidence of the included studies was satisfactory or good but not excellent based on the NHMRC criteria [41].

### Data synthesis and meta-analysis

After the extraction of data and generation of contingency tables, we determined the odds ratios corresponding to each contingency table. Data synthesis yielded sufficient data to conduct pooled-analyses of HHV1-5. The results of meta-analysis, along with corresponding measures of heterogeneity, are presented below in Table 4.

**Table 4:** Overall effect sizes organized by viral species.

Viral Species	Study	Determinant	Cases		Controls		logOR^c^	95% CI (LB, UB)	Pooled logOR (95%CI) (p-value)	*I*^2^	Cochran's Q (p-value)
			+	-	+	-					
HSV1 (HHV1)	[49]	IgG Abs	7	0	8	1	2.6471	0.0931, 75.2901	(L): 1.70 (0.81–3.55) (p = 0.1908); (H): 1.93 (0.91–4.08) (p = 0.1262)	(L): 7.20%; (H): 13.13%	(L): 9.6985 (p = 0.4053); (H): 10.6907 (p = 0.3221)
	[50]	IgG Abs	20	1	18	3	3.3333	0.3176, 34.9901			
	[51]	IgE Abs	7	3	2	8	9.3333	1.1934, 72.9934			
	[52]	Viral DNA	0	7	5	17	0.2121	0.0104, 4.3416			
	[54]	IgG Abs	49	110	9	17	0.8414	0.3506, 2.0190			
	[56]^a^	Viral DNA	1	27	0	100	10.9636	0.4344, 276.6976			
	[56]^a^	IgG Abs	28	0	47	3	4.2000	0.2092, 84.3096			
	[57]	Viral DNA	25	0	30	7	12.5410	0.6827, 230.3673			
	[58]	Viral DNA	2	20	0	11	2.8049	0.1237, 63.5905			
	[59]	Viral DNA	0	11	0	11	1.0000	0.0182, 54.8340			
	[61]	Viral DNA	0	10	0	7	0.7143	0.0127, 40.2042			
HSV2 (HHV2)	[50]	IgG Abs	4	17	4	17	1.0000	0.2143, 4.6663	1.44 (0.47–4.47) (p = 0.5545)	45.40%	14.68 (p = 0.0662)
	[51]	IgE Abs	7	3	2	8	9.3333	1.1934, 72.9934			
	[52]	Viral DNA	0	7	0	22	3.0000	0.0546, 164.8072			
	[54]	IgG Abs	21	138	9	17	0.2874	0.1135, 0.7280			
	[56]^b^	Viral DNA	0	28	0	100	3.5263	0.0684, 181.68			
	[57]	Viral DNA	25	0	30	7	12.5410	0.6827, 230.3673			
	[58]	Viral DNA	0	22	0	11	0.5111	0.0095, 27.4591			
	[59]	Viral DNA	0	11	0	11	1.0000	0.0182, 54.8340			
	[61]	Viral DNA	0	10	0	7	0.7143	0.0127, 40.2043			
VZV (HHV3)	[50]	IgG Abs	20	1	21	0	0.3178	0.0122, 8.2574	L: 2.03 (0.32–12.81) (p = 0.4548); H: 3.29 (0.54–20.06) (p = 0.1972)	L: 60.50%; H: 61.70%	L: 12.67 (p<0.05); H: 13.04 (p<0.05)
	[52]	Viral DNA	0	7	4	18	0.2741	0.0131, 5.7439			
	[54]	IgG Abs	103	56	0	26	97.0885	5.8067, 1623.3222			
	[56]^a^	Viral DNA	2	26	0	100	18.9623	0.8834, 407.0301			
	[56]^a^	IgG Abs	28	0	50	0	0.5644	0.0109, 29.2165			
	[58]	Viral DNA	0	22	0	11	0.5111	0.0095, 27.4591			
	[59]	Viral DNA	0	11	0	11	1.0000	0.0182, 54.8340			
	[61]	Viral DNA	7	3	1	6	14.0000	1.1352, 172.6502			
EBV (HHV4)	[51]	IgE Abs	6	4	3	7	3.5000	0.5492, 22.3045	1.70 (0.50–5.81) (p = 0.3949)	0.00%	1.0603 (p = 0.5885)
	[54]	IgG Abs	3	156	0	26	1.1853	0.0595, 23.6108			
	[61]	Viral DNA	4	6	3	4	0.8889	0.1252, 6.3105			
CMV (HHV5)	[48]	Viral DNA	7	2	0	9	57.0000	2.3614, 1375.8509	DL: 3.40 (1.33, 8.68) (p<0.05); REML: 3.65 (1.27, 10.46) (p<0.05)	DL: 9.20%; REML: 22.10%	DL/REML: 8.82 (p = 0.3675);
	[49]	IgG Abs	7	0	8	1	2.6471	0.0931, 75.2901			
	[50]	IgG Abs	15	6	14	7	1.2500	0.3368, 4.6389			
	[51]	IgE Abs	8	2	2	8	16.0000	1.7883, 143.1561			
	[54]	IgG Abs	21	138	0	26	8.2274	0.4833, 140.0512			
	[56]^b^	Viral DNA	0	28	0	100	3.5263	0.0684, 181.6825			
	[58]	Viral DNA	0	22	0	11	0.5111	0.0095, 27.4591			
	[60]	Viral DNA	0	11	0	11	1.0000	0.0182, 54.8340			
	[61]	Viral DNA	1	9	0	7	2.3684	0.0839, 66.8874			

Abs, antibodies, AV, adenovirus; CMV, human cytomegalovirus; ELISA, enzyme linked immunosorbent assay; DL, Der-Simonian Laird (DL) *τ*^2^ estimator; ES, endolymphatic sac; EBV, Epstein-Barr virus; HSV-1/2, herpes simplex virus 1/2; I-A/B, influenza A/B; MV, Measles virus; ns, not-significant; PCR, polymerase chain reaction; RAST, radioallergosorbent test; REML, restricted maximum likelihood *τ*^2^ estimator, SG, Scarpa’s Ganglion.

a: [56] did not report enough individual participant data to compare indirect and direct measures of viral-infection, so separate estimates were made for each method of ascertainment, one corresponding to a lower bound (L) and the other to a higher bound (H). As described in the methods, this was to ensure that individual participant data did not contribute to pooling/overall-effects calculations more than once.

b: In the case of VZV and CMV, [56] ascertained infection using viral nucleic acids as the only determinant and did not report on host antiviral antibodies.

c: In calculating individual and pooled logORs, a Haldane-Anscombe correction (+0.5) was applied to any data tables where contingency tables had zero-counts across cells from both groups.

#### Cytomegalovirus (CMV)

**Fig 2:**
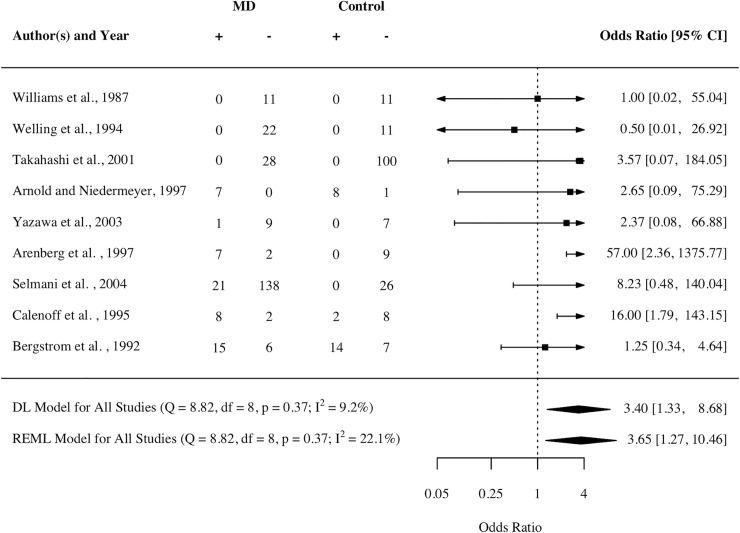
Odds of MD given evidence of CMV infection. The 95% C.I. for the OR obtained in each study down the page is represented by the confidence interval across the page, and the exact values corresponding to the interval are presented on the RHS. The +0.5 values reflect the Anscombe-Haldane corrected data. The overall effects obtained by pooled REML and DL inverse-variance estimators respectively are indicated by the solid black diamonds at the bottommost part of the figure, with the respective Cochran’s *Q* and Higgin’s *I*^2^ heterogeneity statistics presented alongside.

CMV was associated with MD on a narrower logOR interval (DL) of 1.33–8.68 and a wider logOR interval of 1.27–10.46 and neither association displayed any clear sensitivity to high risk of bias studies, nor any between-study heterogeneity beyond that expected by chance. A determinant of CMV-infection was specified within the methodology in 12 of 14 studies, and nine of those reported data that was suitable for inclusion in the meta-analysis. From those 12 studies, under the DL estimator, a significant overall effect size of 3.39 was determined (1.33, 8.65; p<0.05). Thus, the overall effect size was calculated again but under the REML estimator, yielding another significant overall effect size of 3.65 (1.27, 10.46; p<0.05). Heterogeneity was not substantial (Higgin’s *I*^2^) under either DL (9.1%) or REML (22.1%) estimators, nor was heterogeneity significant (Cochran’s *Q*) in either case. Fig 2 displays the results of the meta-analysis of CMV, and thus presents logOR values for each of the nine individual studies and both overall summary measures (DL and REML).

#### Herpes Simplex Virus-1 (HSV-1)

No association was found between HSV-1 and MD, and there was no heterogeneity between the pooled studies beyond that expected by chance. A determinant of HSV-1-infection was specified within the methodology of 13 of 14 studies, and 11 of those reported data that was suitable for inclusion in the meta-analysis. From those 11 studies, two overall effect sizes were obtained under the DL estimator as [56] reported both direct and indirect ascertainments of viral-infection, with effect sizes (logOR (95% C.I.)) of 1.70 (0.81–3.55) and 1.93 (0.91–4.08) according to the inclusion of the indirect and direct ascertainments respectively, thus neither result was significant. Higgin’s *I*^2^ was not substantial under both the high and low DL estimates (7.2% and 15.81%), and Cochran’s *Q* was not-significant in both cases.

#### Herpes Simplex Virus-2 (HSV-2)

No association was found between HSV-2 and MD, and there was substantial heterogeneity beyond chance effect which was unable to be accounted for by the methodological category of ascertainment (direct as opposed to indirect). A determinant of HSV-2-infection was specified within the methodology of 11 of 14 studies, and nine of those presented data that was adequate for pooling, contributing to a non-significant overall effect size under the DL estimator (logOR (95% C.I.)) of 1.44 (0.47–4.47). Higgin’s *I*^2^ was substantial (45.40%) leading to sub-group analysis, while Cochran’s *Q* was not significant. Using the nature of ascertainment (serological or direct) as a moderator in mixed-effects analysis, 10.77% of the overall heterogeneity was accounted for, however no significant overall effect was identified in either subgroup.

#### Varicella Zoster Virus (VZV)

No association was found between VZV and MD, and there was substantial heterogeneity beyond chance effect which was unable to be accounted for by the methodological category of ascertainment (direct as opposed to indirect). A determinant of VZV-infection was specified within the methodology of 10 of 14 studies, and 10 of those presented data that was adequate for pooling. Under the DL estimator (logOR (95% C.I.)), the overall effect size was, as determined by viral nucleic acids, 2.03 (0.32–12.81) (ns), and as determined by host antiviral antibodies, 3.29 (0.54–20.06) (ns). Higgin’s *I*^2^ was substantial (51.90%) and Cochran’s *Q* was significant (14.55, p<0.05), and as such sub-group analysis was performed. Using the nature of ascertainment (serological or direct) as a moderator in mixed-effects analysis, none of the overall heterogeneity was accounted and thus no significant overall effect was identified in either subgroup.

#### Epstein-Barr Virus (EBV)

No association was found between EBV and MD, and there was no heterogeneity between the pooled studies beyond that expected by chance. A determinant of EBV-infection was specified within the methodology of five of fourteen studies, and three of those presented data that was adequate for pooling. Under the DL *τ*^2^ estimator (logOR (95% C.I.)), the overall effect size was 1.70 (0.50–5.81), and thus not significant. Higgin’s *I*^2^ was not substantial (0.00%), nor was Cochran’s *Q* significant.

#### Other viral species

Besides those viral species mentioned above, a determinant of infection by AV was specified in three studies while determinants of infection by MV, I-A/-B and any other viral species were each assessed in two studies. However, the reporting of data was not adequate for determination of ORs.

### Sensitivity analyses

**Fig 3:**
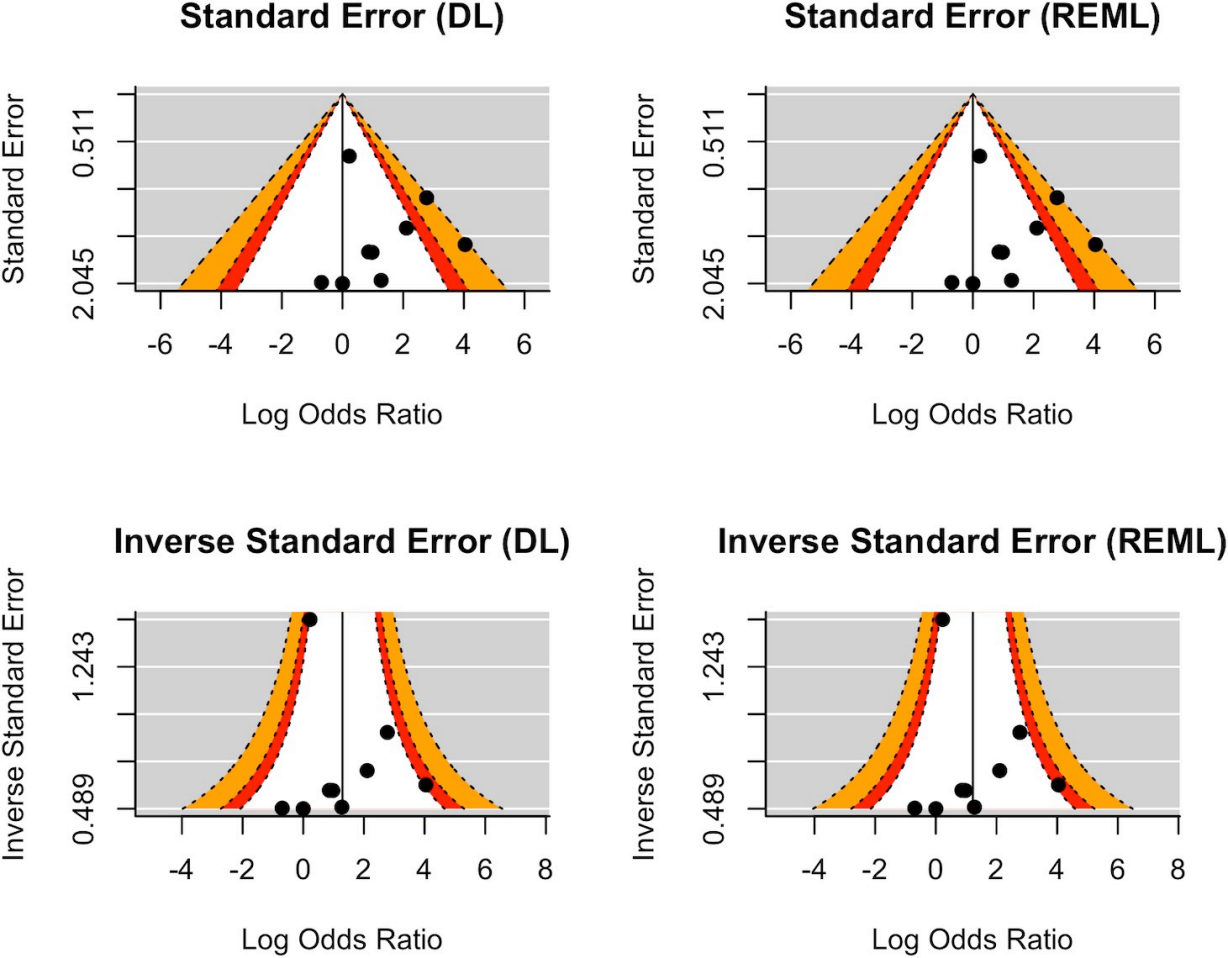
Sensitivity of the relationship between MD and CMV-infection to leave-one-out analyses. The overall effect size(s) obtained by omitting any particular study is indicated in a descending (non-cumulative) manner, with the study omitted indicated on the y-axis by (¬), and the respective effect sizes (log(OR)) presented along with 95% C.I. on the RHS. The overall effect obtained prior to the omission of any study is indicated by the bottommost light grey diamond. A: Sensitivity analysis (DL). B: Sensitivity analysis (REML).

To assess the sensitivity of any reported associations to particular studies, leave-one-out analyses were performed and are presented in Fig 3. Leave-one-out analyses did not reveal any clear sensitivity of the association between CMV and MD to studies that could pose a high risk of bias, however certain studies were required for the significance of the pooled effect. Under the DL estimator, when Calenoff (1995; NOS rating of 3/4-2/2-3/3) was excluded, the overall effect (logOR (95% C.I.)) was not significant 2.33 (0.93, 5.87). The omission of any other study retained the significance of the DL estimated overall effect size. Under the REML estimator, the exclusion of either Calenoff et al. (1995; NOS rating of 3/4-2/2-3/3) or Arenberg et al. (1997; NOS rating of 2/4-0/2-3/3) yielded non-significant overall effect sizes (logOR (95% C.I.)) of 2.54 (0.91, 7.10) and 2.68 (0.99, 7.25) respectively. The omission of any other study retained significance of the REML estimated overall effect size.

### Assessment of publication bias with regards to the CMV-MD association

**Fig 4:**
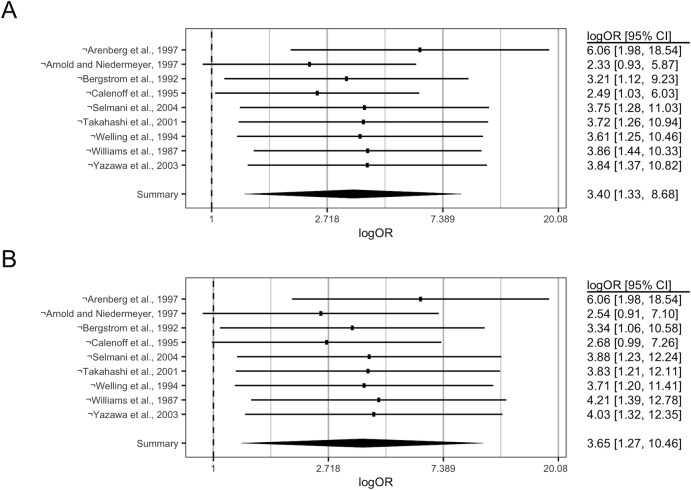
Distribution of studies by power around the measured and null relationship between MD and CMV-infection. In each plot, the white, orange, red, and grey-striped regions represent p>0.1, 0.05<p<0.1, 0.01<p<0.05 and p<0.01 respectively. The y-axes correspond to the standard error, variance, or the inverse of either, while the x-axes correspond to the logarithmically transformed effect size, in this case measured as an OR. The vertical black reference lines are centered on zero for the standard error and variance funnel plots, while they are centered on the estimate effect size for the inverse funnel-plots. The left-hand column pane presents the plots for the DL estimator, while the right-hand column pane presents the plots for the REML estimator.

In attempting to gauge any outstanding publication bias, studies which ascertained evidence of CMV-infection were subject to funnel-plot analyses, themselves summarised in Fig 4. The funnel-plot analyses did not indicate any small-study biases through quantitative assessment, while visual inspection suggested that studies were clustered towards lower power in a non-linear fashion, but overall the power of the funnel-plot analyses was severely limited by the small number of studies (<10). Duval and Tweedie’s trim and fill analysis for data imputation of missing samples was not significant in either the DL (SE = 1.9045) or REML (SE = 1.8356) estimators for the pooled OR of MD given CMV-infection. Egger’s regression test for funnel plot asymmetry was not significant under either DL (z = 0.3476, p>0.05) or REML (z = 0.1467, p>0.05) estimators for CMV overall effects. Moreover, there were no remarkable qualitative findings of left-right asymmetry upon manual inspection of the funnel plots, nor were there obvious differences between the DL and REML plots. The inverse standard error plots revealed a (qualitatively discernible) coalescence of studies towards a lower power.

## Discussion

### Overall findings

We identified 14 case-controlled studies of past or present viral-infection in MD and from these we found a small association between CMV and MD, found no association between other *Herpesviridae* and MD, and encountered insufficient data to quantify relationships between any non-*Herpesviridae* species and MD.

The characteristics of the included studies were diverse. Data availability was comparably higher for CMV than most other viral species. With regards to study quality, the overall mean NOS ascertainment was 5/9, with our a priori selection criteria ensured all studies employed molecularly-verified, case-controlled diagnostics. All of our included studies were level III in the NHMRC evidence hierarchy. On the other hand, none of the included studies conducted a priori power analyses nor justification for non-1:1 sampling in small (*n*<20) studies. Furthermore, selection definitions of both MD and controls varied between studies, and justifications thereof were not always clear. In particular, there was a less than ideal quality of reporting with regards to the health status of control group participants. The average age of the participants (52.6 years) was determined from a sub-set of the included studies due to reporting discrepancies, however this value reflects other epidemiological accounts of MD. Thus, overall, we judge that the studies consisted in a moderate-, but not high-quality evidence base.

Despite including determinants of viral-infection that were not specific to any particular time-scale or state of infection (active or latent), no heterogeneity between pooled studies beyond chance was present for CMV, HSV-1, or EBV, lowering the likelihood that the association found between CMV and MD is an artefact of pooling. Significant heterogeneity as indicated by Higgin’s *I*^2^ for HSV-2 and both Higgin’s *I*^2^ and Cochran’s *Q* for VZV warranted sub-group analyses, for which we had hypothesized a priori that ‘direct’ as opposed to ‘indirect’ ascertainments would account for some of that variance. In the case of HSV-2, the category of ascertainment accounted for some heterogeneity but this reduction in variance was not significant, as was the case for VZV, and thus residual variance for the pooling of these viruses was unable to be explained merely by the collation of both kinds of ascertainment data.

With regards to the association found between evidence of CMV-infection and MD, nine studies were pooled together in determining the overall effect size. For those studies, neither Higgin’s *I*^2^ nor Cochran’s *Q* were substantial or significant, indicating that variance in the pooled-effect size was unlikely to be attributable to heterogeneity (alpha = 0.05). Sensitivity analyses revealed that [51] was necessary for significance under both variance estimators, while [48] was necessary for significance under REML, with those studies respective NOS scores of 8/9 and 5/9 (and level III in the NHMRC hierarchy) suggesting that the reported association was unlikely to be greatly affected by studies introducing a high risk of bias.

In analyses for small-study and other potential publication biases through funnel plots, there were no grounds for data imputation, nor was their evidence for significant funnel-plot asymmetry, although visual inspection of the funnel plots indicated a non-linear clustering of lower powered studies. However, given that only nine studies contributed to the pooled effect sizes determined for the association between CMV and MD, it is unlikely that the funnel-plots presented in this work are powerful indicators of publication biases [62]. Overall, based upon a RE meta-analysis of currently available evidence, CMV shows a modest association with MD, while none of HSV-1,-2, VZV, or EBV are associated, while the question of associations between other viral species and MD is precluded by a lack of data.

### Strengths and limitations

To the best of our knowledge this is the first systematic review and meta-analysis of viral-infection in relation to MD, and beyond characterizing and appraising the literature, we found a modest association between MD and evidence of past or present CMV-infection. We used a broad search strategy in combination with an a priori semi-structured methodology. To minimize study selection bias, we searched four online databases, supplemented with a hand search of the reference lists of studies retrieved from those databases in an attempt to identify any studies not indexed online, from which we found three studies. The methodological quality of the included studies was systematically appraised by use of the NOS, which in conjunction with the NHMRC guidelines, led us to conclude that the modest association found between CMV and MD is probably underpinned by a limited albeit moderate quality evidence base. We included studies from a range of global regions, and this allowed us to address geographical and ethnic bias to some extent. Through our a priori study design, we tried to ensure that eligible studies were those that were comparable at the outset by requiring the use of direct or indirect molecular-determinants in their ascertainment of viral-infection. In fitting a model to our RE meta-analysis, we used inverse-variance estimators of differing sensitivities in order to further gauge the robustness of any overall effects. Further, we performed leave-one-out sensitivity analyses and funnel-plot analyses so as to gauge how our results may have been affected by studies that posed a greater risk of bias, a lack of data or publication bias.

Future studies might improve upon our methods in several regards. One of our main challenges was the clearly limited amount of eligible data, limiting the reliability of the association we have found–this may be rectified in light of further primary data acquired in future studies. Moreover, because the quality of studies from which our data was extracted was not optimal, this limits the reliability of the presently described association. The nature of the described association remains to be uncovered in future works, and it cannot be deduced from this work alone for which subgroup(s) of those with MD this association may or may not be relevant. We did not attempt to investigate the time-course of infections as our primary aim was to assess whether there was an association between evidence of host viral-infection at the time of study or any time previous, and eliciting how the temporality of viral-infection may impact a relation to MD is an important question. Although our study identification was designed to be as broad as possible, we only included articles written in the English language. We selected in only case-controlled studies, which may have placed further constraints on an already limited availability of molecularly verified investigations of viral-infection in MD. Inherent in the limits of a case-control study is that the directionality of any association cannot be concluded from the study data alone, and although methodologies can be optimized to control for confounding factors, the non-randomization of case-control studies was here an unavoidable limitation. Given the range of years, locations, and aims involved in the included studies, we used a RE analysis, but the limited availability of data rendered it difficult to determine precisely to what degree our combining of direct and indirect ascertainments influenced the overall results. Although the quantitative measures of heterogeneity did not indicate substantial or significant heterogeneity with respect to the described association, studies differed qualitatively in several important regards. The included studies appealed to a variety of inclusion criteria, i.e., that did not entirely overlap. The included studies also investigated a range of viruses as a whole, but only a few of these viruses were investigated across most of the studies. Ideally, future meta-analyses would have enough data to measure each overall effect with respect to a more fine-grained constraint on the methodology. Most of the included studies used control groups that had comorbidities, for example, vestibular schwannoma, as opposed to more healthy controls. Finally, despite the use of a semi-structured a priori methodology, our review was not pre-registered with PROSPERO, which is emerging as a preferred method for the undertaking of systematic reviews [63].

### Implications

The idea that viruses, especially those of the *Herpesviridae* family, might contribute to the pathogenesis of MD has remain disputed for some time. The 2008 review of Oliviera and colleagues concluded it to be unlikely that MD had any demonstrable viral etiopathology [15]. On the other hand, contemporaneous work by Gacek concluded that overall, MD simply *is* the manifestation of viral-infection of the inner-ear [18]. In the context of various experimental models of MD thus far, viral pathogens have acted as powerful mediators of EH, itself arguably a crucial component of the MD puzzle [8, 11, 12]. Yet whether such experimental virulence could be of relevance to the clinical setting has remained unclear. Though we did not make an a priori prediction, under our analyses, we found a history of CMV-infection (past or present) is associated with MD. CMV infection, especially in congenital contexts, has been identified as among the most prevalent of the infective etiopathologies of SNHL, with deleterious consequences almost invariably for hearing and often too the vestibular system [64–66]. Moreover, CMV has been suggested to lead to hydropic ear disease, and so too has EH been linked to immune-mediated inner-ear disease [67, 68]. So CMV is probably influential for a variety of pathophysiological processes of the vestibular and cochlear labyrinths that are themselves associated strongly with MD, and thus too in MD CMV-infection may be a factor in facilitating dysregulation of the inner-ear labyrinths, a hypothesis suggested by Arenberg and colleagues [48]. Finally, since CMV is a known pathogen for a variety of immune-mediated etiopathologies of the inner-ear, it is plausible that CMV influences MD via pro-inflammatory states, a hypothesis which integrates with pre-established etiological frameworks suggesting a potential role for infectious pathogens in MD.

Until more is known about the potential influence of CMV on MD, in particular whether this association holds in more rigorous clinical studies with, for one, clinically sub-grouped participants, and for another, viral nucleic acid determinants in each setting, the presently described association is not yet relevant to clinical practice. Trials of antiviral pharmacotherapy have not demonstrated any convincing or reliable efficacy thus far [29]. The diversity of viral involvements described here in terms of both species and natural history (severity and time-course) implies that future trials of antiviral pharmacotherapy for MD should carefully consider the goal of such therapy in relation to the pathophysiology of MD. For one, if only a small proportion of those with MD suffer from a virally-mediated form of the disease, this would need to be taken account in power-analyses or selection measures of future studies. If, on the other hand, viral-infection is merely an exacerbating factor for MD, then this would need to be taken into account in terms of the design of study outcomes. If, else, viral-infection is associated with MD but does not mediate the disease, this might imply a redundancy of antiviral pharmacotherapy. Overall, with regards to the prospects of future trials of antivirals in MD, in making any sort of judgement of *whom* with MD antiviral pharmacotherapy might be indicated, it seems that an initial point of order is to try and better understand any potential epidemiological relationships between viral infection and MD.

Thus, while we have described an association between CMV-infection and MD, the evidence underpinning this association needs to be improved upon in several regards if the association is to be not only elicited but also demonstrated rigorously enough so as to inform clinical practice. On the one hand, further observational studies in the clinical setting, particularly those which employ molecular verification, would probably aid in coming to a more reliable and perhaps more accurate understanding of the epidemiological aspect of this viral association. In particular, any future clinical studies should seek to more rigorously define selection criteria for the case and control groups, improve the clarity and consistency of reporting of the qualitative characteristics of both types of groups, and provide more thorough justifications of whether or not individual participants could be considered to be ‘healthy’, or at least not suffering from any condition that may have confounded the association found in the present study. Beyond the clinical setting, experimental investigations would be of chief importance for understanding how CMV and other viral pathogens might influence or else relate to the pathophysiology of MD. With regards to experimental investigations of viral-infection in MD-models, the beginnings of such investigations have been conducted, although such investigations are particularly difficult given the lack of understanding of MD in general [69–72]. In the last decade or so, models of particular components of MD pathophysiology have become more accessible due to increased efficiency and cost-effectiveness afforded by simple genetic models such as that of the Phex mouse for modelling EH [73]. The increased availability of such models affords the potential for an increased range of analyses of the potential consequences of viral-infection of the inner-ear in the context of MD.

## Conclusion

Here, we systematically reviewed evidence of past or present viral-infection in MD from case-controlled observational molecular studies; we did not restrict our analyses to particular sub-groups of MD or particular viral species. We identified a 14-study body of evidence, from which we found a modest association whereby the odds of MD were greater given evidence CMV-infection. However, the amount of data used to calculate this association was limited and this finding would in future need to be supported by more evidence, preferably too of a higher quality. Other viruses such as influenza-A, -B, and adenovirus have been investigated in relation to MD, but such data was too limited for meta-analysis.

## Supporting information

S1 Table PRISMA checklist. From: Moher D, Liberati A, Tetzlaff J, Altman DG, The PRISMA Group (2009). Preferred Reporting Items for Systematic Reviews and Meta-Analyses: The PRISMA Statement. PLoS Med 6(7): e1000097. doi:10.1371/journal.pmed1000097.
(DOC)

S2 Table MOOSE checklist. From: Stroup DF, Berlin JA, Morton SC, et al, for the Meta-analysis Of Observational Studies in Epidemiology (MOOSE) Group. Meta-analysis of Observational Studies in Epidemiology. A Proposal for Reporting. JAMA. 2000;283(15):2008–2012. doi: 10.1001/jama.283.15.2008.
(DOC)
