# Correction: Are viral-infections associated with Ménière’s Disease? A systematic review and meta-analysis of molecular-markers of viral-infection in case-controlled observational studies of MD

**DOI:** 10.1371/journal.pone.0226643

**Published:** 2019-12-12

**Authors:** Nicholas John Dean, Christopher Pastras, Daniel Brown, Aaron Camp

The images for Figs 3 and 4 are incorrectly switched. The image that appears as Fig 3 should be Fig 4, and the image that appears as Fig 4 should be Fig 3. The figure captions appear in the correct order.

**Fig 3:**
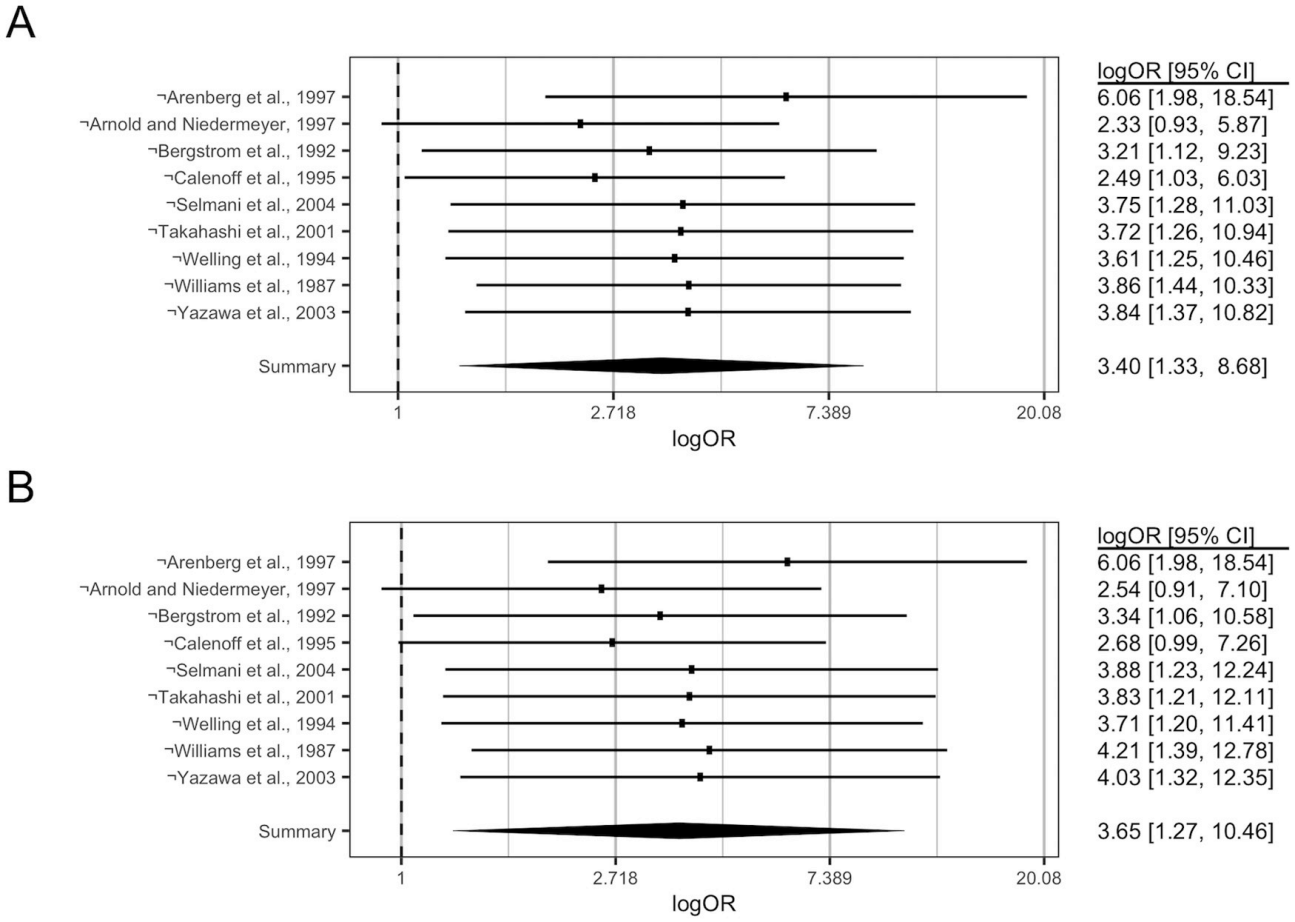
Sensitivity of the relationship between MD and CMV-infection to leave-one-out analyses. The overall effect size(s) obtained by omitting any particular study is indicated in a descending (non-cumulative) manner, with the study omitted indicated on the y-axis by (¬), and the respective effect sizes (log(OR)) presented along with 95% C.I. on the RHS. The overall effect obtained prior to the omission of any study is indicated by the bottommost light grey diamond. A: Sensitivity analysis (DL). B: Sensitivity analysis (REML).

**Fig 4:**
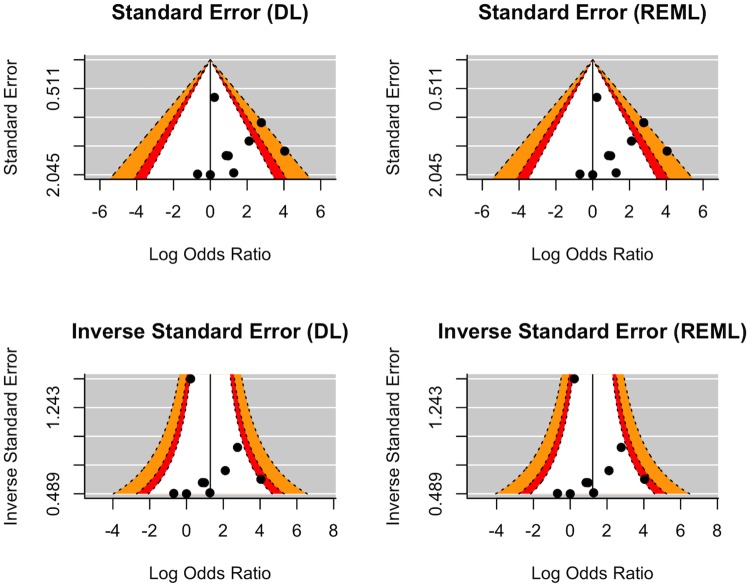
Distribution of studies by power around the measured and null relationship between MD and CMV-infection. In each plot, the white, orange, red, and grey-striped regions represent p>0.1, 0.05<p<0.1, 0.01<p<0.05 and p<0.01 respectively. The y-axes correspond to the standard error, variance, or the inverse of either, while the x-axes correspond to the logarithmically transformed effect size, in this case measured as an OR. The vertical black reference lines are centered on zero for the standard error and variance funnel plots, while they are centered on the estimate effect size for the inverse funnel-plots. The left-hand column pane presents the plots for the DL estimator, while the right-hand column pane presents the plots for the REML estimator.
